# Peripheral metabolism informs on future cognitive decline and development of Alzheimer’s disease in population at risk

**DOI:** 10.1002/alz.089802

**Published:** 2025-01-03

**Authors:** Nuanyi Liang, Kwangsik Nho, John W. Newman, Matthias Arnold, Kevin Huynh, Peter J Meikle, Kamil Borkowski, Rima Kaddurah‐Daouk, Alzheimer’s Gut Microbiome Project Consortium (AGMP), the Alzheimer’s Disease Metabolomics Consortium, for the Alzheimer’s Disease Neuroimaging Initiative

**Affiliations:** ^1^ University of California, Davis, Davis, CA USA; ^2^ Indiana University School of Medicine, Indianapolis, IN USA; ^3^ United States Department of Agriculture, Agriculture Research Service, Davis, CA USA; ^4^ Duke University, Durham, NC USA; ^5^ Helmholtz Zentrum München, German Research Center for Environmental Health, Neuherberg Germany; ^6^ Baker Heart and Diabetes Institute, Melbourne, VIC Australia; ^7^ La Trobe University, Melbourne, VIC Australia

## Abstract

**Background:**

Peripheral metabolic health status can reflect and/or contribute to the risk of Alzheimer’s disease (AD). Peripheral metabolic health status can be indicated by metabolic health markers, such as inflammatory biomarker glycoprotein acetyls (GlycA) and specific components of lipoproteins (e.g., triacylglycerol of high‐density lipoprotein). However, it is unclear if the relationship between peripheral metabolism and AD‐related markers is heterogenous among diverse populations and throughout the disease progression.

**Methods:**

Utilizing Alzheimer’s Disease Neuroimaging Initiative data, we determined whether baseline plasma GlycA can inform on cognitive and brain structural changes among sub‐populations with different diagnosis status. Furthermore, correlation analyses were performed between blood metabolomics and cerebrospinal fluid (CSF) proteomics data in sub‐populations with different diagnosis status or different mild cognitive impairment (MCI)/AD outcomes in 3 years.

**Results:**

GlycA was elevated in AD patients compared to cognitively normal participants. Baseline GlycA level was associated with executive function decline at 3‐9 year follow‐up in participants diagnosed with late mild cognitive impairment (LMCI) at baseline, with similar but not identical trends observed in the future decline of memory and entorhinal cortex volume. In addition, peripheral metabolomics signatures of CSF proteomics were well‐distinguished between cognitive normal participants and AD patients. Moreover, different peripheral‐central metabolic connection was also observed in MCI‐AD converters vs. MCI‐MCI non‐converters across 3 years follow up.

**Conclusion:**

Peripheral inflammation was linked to future cognitive decline and brain structural atrophy for population at risk. In addition, peripheral metabolomics‐CSF proteomics correlation reveals distinguishing peripheral‐central connection patterns in AD patients as well as MCI participant soon to develop AD in 3 years. Findings here point to peripheral systemic inflammation and metabolic health in general as risk factors in AD development, pointing to therapeutic intervention related to periphery metabolic health for patients at risk.

